# Unmet healthcare needs, health outcomes, and health inequalities among older people in China

**DOI:** 10.3389/fpubh.2023.1082517

**Published:** 2023-06-15

**Authors:** Lili Wu, Qin Liu, Rao Fu, Jia Ma

**Affiliations:** School of Economics and Management, China University of Petroleum-Beijing, Beijing, China

**Keywords:** unmet healthcare needs, health outcomes, older people, the CHARLS, urban-rural duality

## Abstract

**Objective:**

This study examines whether the experience of unmet healthcare needs in a large sample of Chinese adults aged 60 and over is associated with adverse health outcomes, and how this association varied across needs related to health conditions.

**Study design:**

The 2013 wave of the China Health and Retirement Longitudinal Study is examined. We adopted latent class analysis to identify groups based on health conditions. Then in each identified group, we examined the extent to which unmet needs were associated with self-rated health and depression. To understand the channels through which unmet needs adversely affected health outcomes, we examined the impact of unmet needs attributed to various factors.

**Results:**

Compared to the mean, experiencing unmet outpatient needs is associated with a 3.4% decrease in self-rated health, and people are twice as likely to have depression symptoms (OR = 2.06). Health problems are even more severe when inpatient needs are not met. The frailest people are most affected by affordability-related unmet needs, while healthy people are most affected by unmet needs attributable to availability.

**Conclusion:**

To tackle unmet needs, direct measures for particular populations will be required in the future.

## 1. Introduction

Unmet healthcare needs, defined as the difference between the healthcare services judged necessary and the actual services received (1), represent a measure of access to healthcare. Those engaged in theoretical research in developed countries have offered several explanations of unmet healthcare needs, including deficiencies in the health system, such as a shortage of healthcare services, and the particular circumstances of the individuals seeking care, such as affordability of services, mild illness, inconvenient traffic, no available treatment, and lack of time (1–5). Recently, some of the focus on unmet needs has shifted to the health consequences of insufficient utilization of healthcare. Evidence suggests that those who forego care may subsequently experience a deterioration in their health status (6–8).

There are three knowledge gaps in recent studies of unmet healthcare. First, research about unmet healthcare needs has mainly focused on the US and European countries. However, perhaps because of disparities in national health and social care systems, there is substantial variation in the prevalence of unmet healthcare needs across countries. Indeed, differences in healthcare systems prevent useful comparisons between US (or European) studies and Asian studies. As for China, it is well known that its dual economy and society have produced a distinct welfare system, under which its health system features an “urban–rural duality.” Although by 2011 China had managed to achieve near-universal health insurance coverage (9, 10), inequalities and discrimination that stem from *hukou* restrictions still exist and exacerbate social differentiation. With different benefit packages provided to urban and rural *hukou* holders (11), fragmented health insurance schemes are suggested to be inextricably linked to inequity in healthcare access. It has not yet been investigated whether the inequity has led to unequal health outcomes associated with access to healthcare services.

Second, the older population, which has additional disabilities, physical illness, and social needs, is more vulnerable than younger generations (12). In China, the 2013 National Health Services Survey showed that up to 27% of people aged 60 and above have been affected by not receiving needed healthcare. Despite this worrisome result, research on the unmet healthcare needs of China's older population has received little attention. In China, older people who are poor live primarily in rural areas, hold rural residence registration (*hukou*), usually experienced lower nutrition during childhood, and in many cases had high exposure to natural and political upheavals, such as the Great Famine (1958–1962) (13). These adverse conditions in life experience and resources are intrinsically linked to disadvantages in late life outcomes, including health. The subgroups of older people who have been affected by these conditions often have greater care needs because of their health vulnerability. In contemporary China, these subgroups are most likely to experience unmet needs because of poverty, an unbalanced allocation of medical resources, or a lack of efficient insurance (14). The treatment that they currently are forgoing in their old age could result in even worse health outcomes and more depressive symptoms. These facts mean that the Chinese data that we examine are well suited to testing the hypothesis that older adults who are otherwise equal in terms of unmet needs might be confronted with different results regarding their health level. Nevertheless, the relationship between health outcomes and the unmet healthcare needs of older people—and how the association varies across vulnerable population subgroups—remains unknown.

Third, it is important to recognize that unmet needs attributable to different circumstances lead to differential health outcomes. According to the WHO's World Health Survey 2015 (15), more than 60% of older people in China have unmet healthcare needs due to economic barriers, whereas, in developed countries, the corresponding number is < 16%. Moreover, evidence shows that in China, most older people live in remote rural areas, where the lack of high-quality medical personnel and the low efficiency of medical services leave much of the population with health service utilization shortages (16). Complicating matters, adult children, who are the principal supporters of the rural older poor, tend to migrate to urban areas, leaving their older relatives behind. Consequently, the health of rural “empty nesters” often deteriorates. They often forego needed care because of high medical expenses, the low efficiency of medical services, or the lack of younger family members, but not because they have “mild symptoms” or make individual choices (“felt would be inadequate”, “too busy”, “disliked doctors”, or “decided not to seek care”). To secure the needed targeted interventions, it is essential that policy makers understand both the potential causes of unmet needs and the channels through which unmet healthcare needs affect health. Yet to date, no studies have investigated how unmet needs that can be attributed to various factors are associated with health outcomes and how an effective strategy to satisfy the need for rural healthcare for older people can be developed.

This paper attempts to bridge these research gaps. Empirically, we apply the 2013 China Health and Retirement Longitudinal Study (CHARLS) to capture evidence on health utilization gaps among people aged 60 and above. The goal of this paper is to investigate the association between unmet healthcare needs and health outcomes among older people in China. In this study, health outcome includes both functional impairments and multimorbidity, common conditions in old age, as well as mental disorders like depression. We test the following hypotheses:

1. The group most adversely affected by unmet healthcare needs is older people who have the frailest health conditions. Thus, unmet needs disproportionately concentrate among vulnerable groups, which might lead to more serious health inequalities.
2. In older people, unmet needs attributable to various factors might produce different situations with regard to health outcomes and the meeting of existing needs.

In the following section, the data setting and analytical strategies are discussed. Section 3 presents the results, while Section 4 examines policy implications.

## 2. Data and methods

### 2.1. Data source and study population

We used data from the 2013 wave of the China Health and Retirement Longitudinal Study (CHARLS), a broad-purposed social science and health survey of residents aged 45 or above in continental China. As a high-quality nationally representative survey, CHARLS 2013 adopted multi-stage stratified Probability Proportional to Size (PPS) sampling and covered 18,621 individuals scattered over 450 villages/urban communities in 28 provinces. The survey was devoted to recording major elements of health status, healthcare, health-related behaviors, and lifestyle as well as rich information on demographic background and demographic characteristics. In the current study, we selected a total of 7,659 individuals aged 60 and above who responded to healthcare-related questions and their covariates.

### 2.2. Measurements

#### 2.2.1. Health profiles

This study employed 10 health indicators to assess heterogeneous health profiles. These indicators measure three dimensions of health: physical conditions, functional disabilities, and psychological status. Indicators describing physical conditions contained chronic conditions and sensory impairments. Six self-reported diseases were identified as chronic conditions (yes/no): hypertension, liver diseases, heart problems, stroke, kidney diseases, and arthritis or rheumatism. Sensory impairments (yes/no) were measured based on self-declared hearing and vision limitations. Functional disabilities refer to seven activities of daily living (ADLs) and six instrumental ADLs (IADLs). Those requiring help in any one of the ADLs or IADLs were defined as “with difficulties.” Activities “with difficulties” or with “no difficulty” were adopted to measure ADLs, while the measure of IADLs was defined as 0 activities, 1–3 activities, or 4 or more activities “with difficulties.” Psychological status was evaluated based on cognitive limitations. Cognitive limitations were measured using 12-item scores. Answers about the date (year, month, and day), day of the week, and season of individual interviews were recorded, as were the results of serial subtractions of 7 from 100 (up to five times); each correct answer was scored 1 point. The other two scores were obtained based on (1) no use of paper, pencil, or any other aid for number subtractions and (2) the ability to reproduce a picture of two overlapped pentagons. Cognitive limitations (yes/no) were defined by a score < 7.

Comparisons of associations between health outcomes and unmet needs among older individuals that do not account for previously accumulated health conditions might produce biased assessments because individuals face different levels of need. To overcome this limitation, we used a latent class analysis (LCA) to identify subgroups of older adults on the grounds of heterogeneous health conditions, and these were identified as healthcare need factors. The LCA was based on the 10 dichotomous measures of health indicators discussed above. The latent class analysis provided additional information by identifying, in terms of patterns of health conditions and needs experienced, underlying subgroups that are mutually exclusive and differ qualitatively.

#### 2.2.2. Major dependent variables

We used self-rated health and depression as our health outcomes. Self-rated health was coded from 1 to 5 to indicate excellent to very poor health status. Depression (yes/no) was measured by the Center for Epidemiologic Studies 10-item scores (CES-10), which have been widely used to measure depressive symptoms in older adults. The CES-10 is a short-form scale that includes 10 items with response options that range from 0 to 4 (0 = Rarely or none of the time; 1 = Some or a little of the time; 3 = Occasionally or a moderate amount of the time; and 4 = Most or all of the time). The total score ranges from 0 to 30; a higher score indicates a higher level of depression. Respondents with a score above 10 were identified as having depressive symptoms.

#### 2.2.3. Unmet healthcare needs

In our data, respondents were asked whether or not they ever needed healthcare but did not receive the needed services. In total, we identified two types of unmet healthcare needs:

- Unmet needs for a doctor's visit: It is defined by the respondents' providing “yes” to the question, “in the past month, was there ever a time when you were ill but did not visit any medical facilities or medical providers for outpatient care?”
- Unmet needs for hospitalized treatment: It is defined by giving “yes” when asked, “in the past year, was there ever a time when you were not hospitalized after receiving a doctor's recommendation for hospital admission?”

The reasons were recorded, and unmet needs were grouped into four categories: unmet needs due to financial constraints; unmet due to availability (lack of time, inconvenient traffic, no available treatment, and poor healthcare service); unmet due to mild symptoms; and unmet due to other reasons not specified. These survey responses provided possible targets for policy actions (3).

#### 2.2.4. Other variables

Information about the sociodemographic characteristics of participants was collected by referring to previous research and the human capital model. This study includes three major predictors of change in an individual's health stock studied in the pure investment model: age, income, and education. Following the findings of previous studies—that the oldest of the older adults tend to have different healthcare needs—we identified two age groups: 60–69 years and 70 years and above (12). Individual income was classified into four levels: lower than 1,000 yuan; 1,001–4,500 yuan; 4,501–9,000 yuan; and higher than 9,000 yuan. Education level was categorized as illiterate, literate or primary school, and junior high and above.

Following the main social health insurance schemes in China, a variable for health insurance was grouped into Urban Employee Basic Medical Insurance (UEBMI), Urban Resident Basic Medical Insurance (URBMI), the New Rural Cooperative Medical Scheme (NRCMS), and other or more than two insurance programs. These schemes target different populations on the grounds of individuals' residence registration (*hukou*) and/or employment status.

To capture the feature of “urban–rural duality” in the health system, the residence areas where respondents live were categorized into urban and rural areas.

We also used province GDP per capita to capture regional disparity between provinces.

### 2.3. Analytical strategy

Our statistical analysis investigated the prevalence of unmet healthcare needs as well as unmet needs due to categorized reasons. In the previous LCA step, we summarized the characteristics of each study subgroup obtained.

Next, we examined the associations between unmet healthcare needs and health outcomes, while controlling for potential confounding variables. To understand the channels by which unmet healthcare needs adversely affected health outcomes, we examined the impact of various barriers to healthcare. Throughout this article, we use ordinary least squares for continuous dependent variables and the binary logistic model for binary dependent variables. In addition to analysis among all groups, we perform a subgroup analysis to target health disparities.

## 3. Results

### 3.1. Health classes of older people

All 12 health indicators were selected in the LCA model. Comparing models with 2 to 6 classes, we found that the model with 4 classes obtained the lowest BIC (111,825.6) and a relatively lower aBIC (111,606.37). Therefore, in this study, we applied the 4-class LCA model. The labels of health classes were defined in accordance with conditional health indicator probabilities (Table 1). During the study period, the first class was inclined to have the highest probabilities of hypertension, ADLs and IADLs difficulties, and a higher prevalence of cognitive problems. This health class, therefore, was designated “severely frail.” In contrast, the fourth class was labeled “relatively healthy” because it tended to have relatively lower probabilities of chronic diseases, functional impairments, and cognitive problems. The second class, prone to have the highest probabilities of most chronic diseases, was labeled “chronically ill.” The third class was labeled “incapacitated” both physically and cognitively. Although older people in this class exhibit the lowest probabilities of most diseases, they had the highest prevalence of cognitive problems and higher probabilities of ADLs and IADLs with 1-3 activities.

**Table 1:** Conditional probabilities (%) of health indicators in each CLA of older people.

**Health indicators**	**Severely frail (*N =* 777)**	**Chronically ill (*N =* 2,184)**	**Incapacitated (*N =* 1,485)**	**Relatively healthy (*N =* 3,213)**
Hypertension	0.501	0.465	0.197	0.246
Liver diseases	0.053	0.116	0.010	0.026
Heart problems	0.275	0.357	0.036	0.092
Stroke	0.136	0.055	0.005	0.005
Kidney diseases	0.122	0.179	0.016	0.030
Arthritis or rheumatism	0.546	0.607	0.445	0.265
Sensory limitations	0.252	0.476	0.312	0.578
ADLs difficulties	0.828	0.310	0.239	0.055
**IADLs difficulties**				
0 activity	0.034	0.846	0.598	0.973
1–3 activity	0.679	0.154	0.386	0.027
4 or more activities	0.287	0.000	0.016	0.000
Cognitive disorders	0.667	0.254	0.726	0.152

### 3.2. Statistical analysis

Table 2 reports the prevalence of unmet healthcare needs experienced and the causes of unmet needs in the population as a whole and in the four health profiles. Overall, approximately 14% of older people responded they had experienced an unmet need for a doctor's visit and 8% responded they had experienced an unmet need for inpatient service. In total, 14.6% of old people experienced unmet needs because of financial burdens and 59.2% because they had mild symptoms.

**Table 2:** General characteristics of participants [mean (standard deviation)/frequency].

	**Full sample *N =* 7,659**	**Severely frail *N =* 777**	**Chronically ill *N =* 2,184**	**Incapacitated *N =* 1,485**	**Relatively healthy *N =* 3,213**
**Response**					
**Unmet needs experienced**					
Outpatient	13.86	16.73	16.41	13.36	11.65
Inpatient	8.41	15.18	14.01	6.93	3.64
**Unmet needs, causes**					
Economic reason	14.62	31.54	17.04	14.14	6.68
Availability	6.42	7.69	8.10	4.55	5.35
Mild symptoms	59.15	32.31	52.79	58.59	74.87
Other	19.62	27.69	22.07	22.73	12.83
**Predictors**					
**Age**					
45–64	64.79	44.92	65.38	57.57	72.52
Above 65	35.21	55.08	34.62	42.42	27.48
Gender (% of male)	59.09	63.32	58.52	71.31	52.82
**Marriage status**					
Married/partnered	86.19	77.73	87.00	81.82	89.70
Divorced/separated/widowed	13.08	21.49	12.18	17.03	9.84
Single	0.73	-	0.68	1.14	0.48
**Education**					
Illiterate	30.43	50.06	23.63	57.44	17.83
Literate/primary school	41.51	36.94	46.89	35.01	41.95
Junior high and above	28.06	13.00	29.49	7.54	40.21
**Residence area**					
Rural area	63.09	71.81	63.05	76.30	54.90
Urban area	36.91	28.19	36.95	23.70	45.10
**Individual income**					
< 1,000	34.12	44.92	35.16	45.86	25.37
1,001–4,500	26.17	28.96	25.87	29.63	24.09
4,501–9000	21.19	15.63	21.57	14.95	25.12
Above 9,000	17.94	10.17	16.80	9.29	24.59
**Health insurance**					
NRCMs only	74.32	80.31	73.63	84.71	68.53
UEBMI only	10.12	5.15	11.36	2.69	13.91
URBMI only	4.79	3.60	5.59	2.29	5.70
Other or >2 insurance programs	6.52	5.66	6.64	4.04	7.78
**Self-rated living standard**					
Low	16.62	28.83	21.20	18.72	9.59
Medium	60.80	49.68	61.40	56.83	64.92
High	22.57	21.49	17.40	24.44	25.49
**Province GDP per capita**					
< 35,001	47.67	53.93	45.33	59.19	42.42
35,001–45,000	20.12	19.31	22.99	17.58	19.54
>45,001	32.21	26.77	31.68	23.23	38.03

Meanwhile, significant differences in unmet healthcare needs and sociodemographic characteristics were found among older people who had different health profiles and needs. Unmet outpatient needs varied from 11.7% among relatively healthy people to 16.7 % among their severely frail counterparts, while unmet inpatient needs varied from 3.6 to 15.2%. Not surprisingly, older people, who on the whole have frailer health conditions, were more likely than others to have unmet healthcare needs. Moreover, frailer people had higher probabilities of being older, less educated, living in rural areas, earning a lower income, and having lower living standards. Among the severely frail, more than 55% were aged 70 or above, 63.32% were women, 50.06% were illiterate, and 71.81% were rural residents. In contrast, < 30% of relatively healthy people were over 70, only 17.83% were uneducated, and about half were urban residents.

Our sample shows wide variability in the causes of unmet healthcare needs. Among severely frail people, affordability tended to be the main hurdle, accounting for 31.54% of cases. In contrast, among relatively healthy people, 74.87% identified mild symptoms as the top reason for unmet needs, while only 6.68% experienced economic hurdles.

### 3.3. Unmet healthcare needs and health outcomes

Table 3 summarizes regression results of associations between unmet healthcare needs and health outcomes. All estimated impacts of unmet healthcare needs on outcomes, irrespective of how they were defined, are statistically significant at the 1% level. The impact of unmet needs was adverse: it negatively affected the two direct measures of health. Compared to the mean of self-rated health in the full sample (3.722), experiencing unmet outpatient needs predicted a 3.4% (0.129/3.722) decrease, while non-hospitalization led to a decrease as high as 11.1%. Meanwhile, those having unmet inpatient needs were twice as likely as those having no unmet needs to have depression symptoms (OR = 2.06).

**Table 3:** Results for unmet needs (all causes) and health outcomes.

	**Self-rated health**	**Depression**
**All groups**		
Unmet outpatient needs	0.129^***^ (0.034)	1.478^***^ (0.106)
Unmet inpatient needs	0.421^***^ (0.042)	2.063^***^ (0.187)
**Severely frail**		
Unmet outpatient needs	0.041 (0.075)	1.348 (0.332)
Unmet inpatient needs	0.643^**^ (0.077)	2.236^***^ (0.648)
**Chronically ill**		
Unmet outpatient needs	0.095 (0.076)	1.305^***^ (0.240)
Unmet inpatient needs	0.222^**^ (0.100)	1.622^**^ (0.381)
**Incapacitated**		
Unmet outpatient needs	0.094^**^ (0.053)	1.210 (0.154)
Unmet inpatient needs	0.203^**^ (0.089)	1.271^*^ (0.175)
**Relatively healthy**		
Unmet outpatient needs	0.014 (0.045)	1.224 (0.204)
Unmet inpatient needs	0.118^***^ (0.049)	0.995 (0.221)

All regressions are adjusted for age, gender, marital status, education, residence areas, income level, insurance type, health utilization behaviors, self-rate living standard, and province economic level.

^***^p < 0.01, ^**^p < 0.05, and ^*^p < 0.1.

In subgroup regressions, we observed obvious heterogeneities. Among severely frail older people, despite the insignificant impact of unmet outpatient needs, foregoing needed inpatient care resulted in a 14.5% (0.643/4.43) decline in self-rated health. Among relatively healthy older adults, the decline in self-rated health was as low as 3.7% (0.118/3.21). Not surprisingly, the frailest people experienced the greatest decline. In Table 3, going downward from “Severely frail” to “Relative healthy” people, we find a decreasing severity of the effects. Similarly, descending impacts of unmet needs coincide with improvements in health conditions when depression is used as a health outcome. This result shows that a higher possibility of unmet needs was significantly associated with the presence of depressive symptomatology among severely frail older people and people with high comorbidity. No significant associations between unmet needs and depressive symptomatology were found among those who were relatively healthy. In addition, our results show that foregoing hospitalization has greater negative impacts on health outcomes than outpatient care. Meanwhile, the impacts of foregoing hospitalization, a more expensive care, were more different among groups.

Table 4 summarizes for all groups and the four subsamples the estimated impacts of unmet healthcare attributed to the four reasons for self-rated health and depression. For all groups, using “not experiencing unmet needs” as the reference, unmet needs attributable to various factors all exhibit significant links with negative health outcomes, except that unmet needs due to mild symptoms were not a significant risk factor for poor self-rated health. Unmet needs caused by economic factors show the biggest association with health outcomes. It induced a 12.4% (0.462/3.72) decline in self-rated health from the mean, and it shows an OR of more than 3 (OR = 3.49) for reporting depression. Availability-related unmet needs were the second-biggest threat to older people's health, resulting in a 9.6% (0.359/3.72) decline in self-rated health.

**Table 4:** Results for unmet needs with reasons and health outcomes.

	**All groups**	**Severely frail**	**Chronically ill**	**Incapacitated**	**Relatively healthy**
**Self-rated health**					
Economic reason	0.462^***^ (0.077)	0.586^*^ (0.113)	0.117 (0.092)	0.219 (0.170)	0.184 (0.176)
Availability	0.359^***^ (0.116)	0.267 (0.225)	0.035 (0.133)	0.133 (0.191)	0.718^**^ (0.327)
Mild symptom	0.017 (0.039)	0.101 (0.111)	0.099^*^ (0.054)	0.107^**^ (0.053)	−0.107 (0.089)
Others	0.375^***^ (0.066)	−0.058 (0.119)	0.248^***^ (0.081)	0.148 (0.123)	0.283^**^ (0.139)
**Depression**					
Economic reason	3.498^***^ (0.624)	3.499^***^ (1.877)	2.980^***^ (1.265)	1.983 (0.588)	1.733 (0.697)
Availability	2.574^***^ (0.654)	1.961 (1.571)	3.861^***^ (1.756)	1.619 (0.659)	1.991 (1.423)
Mild symptom	1.212^**^ (0.103)	0.978 (0.348)	1.818^***^ (0.265)	1.081 (0.169)	1.176 (0.231)
Others	1.725^***^ (0.245)	1.394 (0.557)	1.309 (0.491)	1.131 (0.268)	1.327 (0.412)

All regressions are adjusted for age, gender, marital status, education, residence areas, income level, insurance type, health utilization behaviors, self-rate living standard, and province economic level.

^***^p < 0.01, ^**^p < 0.05, and ^*^p < 0.1.

Table 4 also reveals that the effects of unmet needs attributed to different factors varied across groups. Columns two to five report the results for the four subgroups, respectively. Among severely frail older people, the adverse consequences of experiencing unmet healthcare because of economic factors were larger than the consequences arising from reporting unmet needs for any reason. Meanwhile, unmet needs attributed to economic factors affected the frailest group more than any other. In contrast, patterns were quite different among relatively healthy individuals. When economic reasons were not considered (at the level of *p*-value = 0.1), availability-related unmet needs significantly explained adverse health outcomes. In addition, in this group, no unmet needs (due to any factor) were associated with depression. Among relatively healthy people, foregoing care because of lack of time, inconvenient traffic, no available treatment, and poor healthcare service evidently were the biggest threats to health.

Between the two extremes, in the chronically ill and incapacitated groups, unmet needs because of mild symptoms showed significant associations with negative health outcomes in physically or cognitively incapacitated people. Perhaps these people did not recognize that they needed healthcare interventions, or perhaps they were reluctant to seek treatment because they had no company. Neglect of this kind often had a negative effect on the self-rated health of the physically or cognitively incapacitated.

## 4. Discussion

This is the first detailed analysis to discern the effect of unmet health care on health outcomes among older people in China. We reach four principal conclusions. First, the analysis presents strong empirical evidence that not enough access to healthcare services has an adverse impact on current health outcomes. Meanwhile, unmet healthcare needs due to problems of affordability and availability seem to be the two principal obstacles to good health. It seems that health inequity, coming from both the demand and the supply side, still remains a major preoccupation in China.

Second, we find that the adverse effects are not distributed evenly across the population. Therefore, we verify our first hypothesis. In general, older people are the most vulnerable group because they have suffered the most from restrictions on access to healthcare. Nevertheless, we find that huge differences exist even among older people: those with the frailest health conditions, who are the most vulnerable among the vulnerable, are more adversely affected than other counterparts. On average, the most affected people are older and economically poorer, have lower levels of education, and reside in less developed areas, including rural areas. They have accumulated high levels of hardships and adversities because of poverty, starvation, and a lack of medical care in early life. Now, most of them live in remote rural areas, where the lack of higher quality medical personnel and the low efficiency of medical services leave much of the population with health service utilization shortages (16). The vulnerabilities that result from these deprivations have led them to life trajectories conducive to adverse health in later life (13). Therefore, compared to the health status of other groups, their health is likely to be frailer and more likely to be severely affected if they do not receive timely and sufficient needed healthcare services. Scholars concerned about the unmet healthcare needs of older people must also consider the efficiency of the healthcare system and the effectiveness of its mechanism for distributing healthcare benefits.

Third, we find that the effects of unmet needs can be attributed to various factors that vary across groups because of differences in health conditions, thus confirming our second hypothesis. Unmet needs attributable to financial barriers present the most adverse effects among older people with the frailest health conditions. China has achieved near-universal health insurance coverage. This remarkable progress has generated a large body of research that has examined the association between China's health insurance and healthcare received. However, it is not yet clear whether China's health insurance has reduced the possibility of not receiving needed healthcare. Most of those most affected by unmet healthcare needs have been rural *hukou* holders, covered by the New Rural Cooperative Medical Scheme (NRCMS). Compared to the insurance provided to urban residents (URBMI) and urban employers (UEBMI), NRCMS is usually regarded as a more rudimentary type of insurance (11); that is, it has the highest deductibles, the lowest reimbursement rates, and the highest coinsurance rates (17, 18). Many services are not covered or are only partially covered by NRCMS. Given the rising costs of healthcare and the low level of NRCMS insurance coverage, it is not surprising that seniors with vulnerable SES are less likely than their urban counterparts to use healthcare services, despite their vulnerable health status. Even worse, they are more likely to forego needed but expensive inpatient care (such as hospitalization), which is a further blow to their already fragile health.

Tackling unmet healthcare needs requires ensuring that individuals in need receive appropriate healthcare. During the past decades, with the goal of ensuring equal needed healthcare access and healthcare utilization, China has undergone a series of reforms in its healthcare system. However, challenges remain in several areas. Owing to the “urban–rural duality” health system and the fragmented health insurance schemes, the protection of the most vulnerable has yet to be fully realized. To remove barriers for those most vulnerable, policies should place more emphasis on reforming the current healthcare system by redistributing healthcare resources toward rural areas and integrating urban and rural insurance schemes. Although relatively equal access to basic care has been provided in outpatient settings, a more effective and low-cost healthcare system is still needed to protect vulnerable people from high healthcare expenses (19).

This study has two limitations. First, cross-sectional data are used, thus any causal relationships between unmet healthcare needs and deterioration of health status cannot be interpreted. To overcome this limitation, further longitudinal studies are needed. Second, because endogeneities might exist, finding and identifying the consequences of unmet healthcare needs is not easy: unmet needs could be a result of adverse health status, or they could be a cause. While we do not infer causality from our estimates, we have learned something important about the associations between unmet healthcare needs and health outcomes, as well as the degree of association differentials by health.

## 5. Conclusion

The present study, for the first time in China, provides population-representative results for healthcare needs and health outcomes in older individuals aged 60+ years. Regressing health outcome variables on unmet healthcare needs and covariates reveals that their associations are significant, indicating that too much delay in care or not enough access to healthcare during the study period was associated with adverse health outcomes.

Performing additional analyses to examine the differences in the associations between unmet healthcare needs and health outcomes by perceived health status, we observe a significant disparity of associations. According to our results, the severely frail group was affected more frequently and more seriously by unmet healthcare needs than other groups. Those in the severely frail group delayed seeking medical care mainly because of affordability problems. The adverse effects of affordability-related unmet healthcare needs are not distributed evenly across the population. Older people with health conditions and who are frailer are more adversely affected than their healthier counterparts. The economic burdens are greater for those who are frailer in health and more urgently in need of healthcare, suggesting that socioeconomic status might be a cause of disparities in healthcare use. Although a near-universal health insurance system has been established in China to improve the overall accessibility to healthcare, cost barriers have remained a significant obstacle to the most vulnerable population, for whom disadvantages, such as poverty, the lack of care, and poor health conditions, have as a cluster led to even worse results. Measures that combat the presence of unmet healthcare needs could positively impact health, but what is most needed in the future will be direct measures aimed at particular populations.

## Data availability statement

Publicly available datasets were analyzed in this study. This data can be found here: https://charls.charlsdata.com/pages/data/111/zh-cn.html.

## Ethical approval

The data came from the CHARLS, which was approved by the Ethical Review Committee of Peking University, and all participants signed informed consent forms at the time of participation.

## Author contributions

All authors listed have made a substantial, direct, and intellectual contribution to the work and approved it for publication.
